# The Health and Functioning ICF-60: Development and Psychometric Properties

**DOI:** 10.1002/cpp.1909

**Published:** 2014-06-15

**Authors:** V A Tutelyan, S Chatterji, A K Baturin, A V Pogozheva, O N Kishko, S E Akolzina

**Affiliations:** 1 Institute of Nutrition of the Russian Academy of Medical SciencesMoscow, Russia; 2 World Health OrganizationGeneva, Switzerland; 3 Vision International People Group

**Keywords:** ICF, Health and Functioning, Nutrition, Quality of Life, Psychometrics, Population Surveys

## Abstract

**Background:**

This paper describes the development and psychometric properties of the Health and Functioning ICF-60 (HF-ICF-60) measure, based on the World Health Organization (WHO) ‘International Classification of Functioning, Disability and Health: ICF’ (2001). The aims of the present study were to test psychometric properties of the HF-ICF-60, developed as a measure that would be responsive to change in functioning through changes in health and nutritional status, as a prospective measure to monitor health and nutritional status of populations and to explore the relationship of the HF-ICF-60 with quality of life measures such as the World Health Organization WHOQOL-BREF quality of life assessment in relation to non-communicable diseases.

**Methods:**

The HF-ICF-60 measure consists of 60 items selected from the ICF by an expert panel, which included 18 items that cover Body Functions, 21 items that cover Activities and Participation, rated on five-point scales, and 21 items that cover Environmental Factors (seven items cover Individual Environmental Factors and 14 items cover Societal Environmental Factors), rated on nine-point scales. The HF-ICF-60 measure was administered to the Russian nationally representative sample within the Russian National Population Quality of Life, Health and Nutrition Survey, in 2004 (*n* = 9807) and 2005 (*n* = 9560), as part of the two waves of the Russian Longitudinal Monitoring Survey (RLMS). The statistical analyses were carried out with the use of both classical and modern psychometric methods, such as factor analysis, and based on Item Response Theory, respectively.

**Results:**

The HF-ICF-60 questionnaire is a new measure derived directly from the ICF and covers the ICF components as follows: Body Functions, Activities and Participation, and Environmental Factors (Individual Environmental Factors and Societal Environmental Factors). The results from the factor analyses (both Exploratory Factor Analyses and Confirmatory Factor Analyses) show good support for the proposed structure together with an overall higher-order factor for each scale of the measure. The measure has good reliability and validity, and sensitivity to change in the health and nutritional status of respondents over time. Normative values were developed for the Russian adult population.

**Conclusions:**

The HF-ICF-60 has shown good psychometric properties in the two waves of the nationally representative RLMS, which provided considerable support to using the HF-ICF-60 data as the normative health and functioning values for the Russian population. Similarly, the administration of the WHOQOL-BREF in the same two waves of the nationally representative RLMS has allowed the normative quality of life values for the Russian population to be obtained. Therefore, the objective assessment of health and functioning of the HF-ICF-60 could be mapped onto the subjective evaluation of quality of life of the WHOQOL-BREF to increase the potential usefulness of the surveys in relation to non-communicable diseases. © 2014 The Authors. *Clinical Psychology & Psychotherapy*. Published by John Wiley & Sons, Ltd.

**Key Practitioner Message:**

1. The HF-ICF-60 offers a new perspective in measuring change in functioning through changes in lifestyle and diet.
2. The HF-ICF-60 can be combined with the WHOQOL-BREF to map the objective assessment of health and functioning onto the subjective evaluation of quality of life.
3. Combined use of the HF-ICF-60 and the WHOQOL-BREF can be especially useful for national and global monitoring and surveillance of implementation of measures to reduce risk factors of non-communicable diseases and to promote healthy lifestyles and healthy diets.

## BACKGROUND

Non-communicable diseases are the leading causes of mortality and disability and significantly impact the lives of individuals in high-income, middle-income and low-income countries (WHO, 2005). The magnitude of these diseases has reached epidemic proportions, yet the global epidemic can be reversed through strengthening national and global monitoring and surveillance, scaling up the implementation of evidence-based measures to reduce the major risk factors (WHO, 2011a). Global and national declarations recognize that effective non-communicable disease prevention and control require leadership and concerted government action at all levels, giving the highest priority to promoting and supporting healthy lifestyles and healthy diets as levers of improving the nation’s health (RF President Decree, 2012; United Nations, 2011; WHO, 2011b).

Development and implementation of the global health promotion strategies requires a consistent framework for studying health and health-related states. The WHO family of international classifications provides a valuable tool to describe and compare the health and functioning of populations, internationally as well as nationally, permitting comparisons of data within and between populations. As a member of the WHO Family of International Classifications, the International Classification of Functioning, Disability and Health (ICF) (WHO, 2001) provides a conceptual framework for measuring health. The ICF uses as its conceptualisation the biopsychosocial model of disability that represents a synthesis of the medical and social models, rather than a mere adoption of one or the other (Bickenbach, Ustun, Chatterji, & Badley, 1998). The ICF can be used to describe health and health-related states associated with all health conditions; thus, the ICF has universal application. The domains contained in the ICF are described from the perspective of the body, the individual and society in four basic chapters: Body Functions, Body Structures, Activities and Participation, and Environmental Factors (WHO, 2001).

Environmental factors make up the physical, social and attitudinal environment in which people live and conduct their lives (WHO, 2001). These factors can have a positive or negative influence, facilitating or hindering impact, on an individual’s health and functioning. A major factor influencing health is nutrition. Unhealthy diets, insufficient or excessive intakes of certain nutrients, may lead to cardiovascular diseases, digestive system diseases, metabolic disorders and deterioration of quality of life (Tutelyan & Onishchenko, 2009; Tutelyan, Spirichev, Sukhanov, & Kudasheva, 2002).

The main aim of the present paper was to introduce the Health and Functioning ICF-60 (HF-ICF-60), a measure of health and functioning that drew directly from the ICF and included the components as follows: Body Functions, Activities and Participation, and Environmental Factors (Individual and Societal Environmental Factors) (WHO, 2001). The chapters that cover body structures in the ICF were, therefore, not included. It is important to note that the measure builds on other international exercises such as the ICF-based development of the World Health Organization Disability Assessment Schedule, the WHODAS 2.0, which includes the six domains of cognition, mobility, self-care, getting along, life activities and participation (WHO, 2010). In ICF terms, the WHODAS 2.0 focuses more on activities and participation, whereas the new HF-ICF-60 measure was designed also to focus on the assessment of body functions and the impact of environmental factors on them.

The primary purpose behind the development of the HF-ICF-60 was to develop a tool for monitoring and surveillance of implementation of measures to reduce risk factors of non-communicable diseases and to promote healthy lifestyles and healthy diets. Although nutrition is one of the focuses of the measure, the measure has broad use across all the domains of the ICF. This main purpose was achieved through the use of the HF-ICF-60 in the Russian National Population Quality of Life, Health and Nutrition Survey as part of the Russian Longitudinal Monitoring Survey (RLMS), which is a longitudinal general population-based nationally representative survey, held annually from 1992 up to now (see below). Furthermore, there was a need to broaden the coverage of domains across the ICF, in comparison with earlier ICF-based measures, and, additionally, to develop a measure that would be responsive to changes in health and nutritional status and thereby allow the assessment of functioning that would change with health and nutritional status in the general population. Taking into account the status of the RLMS, the inclusion of the HF-ICF-60 in the survey, moreover, has served the purpose of assessing the data obtained as the normative values for the Russian population.

It is important to note, that in addition to drawing on the ICF, the new measure was also designed to parallel the structure of the WHO measures of quality of life, the WHOQOL-100 (WHOQOL Group, 1998a) and the WHOQOL-BREF (WHOQOL Group, 1998b). Both the WHOQOL measures and the new НF-ICF-60 use scales to assess physical and psychological health, social and environmental factors. Nevertheless, the WHOQOL measures assess subjective perception of health in terms of the WHO definition of health and reflect what people ‘feel’ about their health status; hence reflect ‘subjective well-being’, whereas the constructs used in the HF-ICF-60 refer to objective description of health status of the individual (WHO, 2001). Besides, what the НF-ICF-60 does however is expand the range of health parameters measured in the WHOQOL. Therefore, the subjective evaluation of quality of life provided by the WHOQOL measures could be mapped onto the objective assessment of health and functioning of the ICF-based HF-ICF-60 measure.

## METHODS

### Design and Procedure

The HF-ICF-60 pilot version was pre-tested with a sample of 50 healthy and ill respondents to provide preliminary feedback on any problems with wording, any problems with the response scales, any problems with the instructions and respondents’ overall impression of the measure. Following this initial pilot, the HF-ICF-60 was further refined and psychometrically tested. The development pilot testing involved the administration of the HF-ICF-60 pilot form to 500 adult respondents (300 respondents with a disability and 200 healthy respondents). Respondents with a disability were in-patients of the Dietotherapy Clinic of the Institute of Nutrition of the RAMS. Considering that the HF-ICF-60 was administered to monitor health and nutritional status; overweight or obesity, in addition to disability, was a prerequisite for inclusion of a patient in the disability pilot sample. The healthy respondents for the pilot sample were recruited from university students in Moscow.

The field trial of the HF-ICF-60 psychometric properties reported here involved the administration of the measure in the Russian National Population Quality of Life, Health and Nutrition Survey as part of the RLMS on a national stratified multi-stage probability sample representative of all Russia (Swafford, Kosolapov, & Kozyreva, 1999).

The survey was held in two waves, 1-year apart, in October 2004 to January 2005, and followed in October 2005 to January 2006. The data show that the majority of the respondents in the 2004 survey were successfully followed up in 2005, though some additional respondents were collected in 2005 in order to make the numbers up to approximately 10 000. Table 1 shows the distribution of the respondents in the 2004 and the 2005 samples by sex, age and health status (self-report). The profile of non-communicable diseases for those who reported themselves to be ill shows the prevalence of cardiovascular, digestive system and musculoskeletal diseases.

**Table 1:** Characteristics of the survey sample

	The 2004 sample	The 2005 sample
Distribution by sex (%):		
Male	42.8	43.1
Female	57.2	56.9
Distribution by age group in years (%):		
14–19	11.8	11.1
20–29	19.8	20.0
30–39	16.8	17.5
40–49	17.9	17.2
50–59	13.6	14.6
60–69	10.5	9.7
70+	9.3	10.0
No response	0.2	0.0
Distribution by health status (%):		
Healthy	76.0	76.8
Ill	22.1	21.5
No response	1.9	1.7
Total	9807	9560

The HF-ICF-60 questionnaire used the self-administered technique but in the presence of an interviewer. The collected data were entered twice to correct possible input errors.

### Russian Longitudinal Monitoring Survey Sample

The Russian National Population Quality of Life, Health and Nutrition Survey was carried out as part of the RLMS. The RLMS is based on a national stratified multi-stage probability (random) area sample of the Russian population (Swafford *et al*., 1999). The sample was designed to represent both households and individuals residing in those households.

First, a list of 2029 consolidated regions (administrative regions similar to counties) was created from which to draw primary sample units (PSUs). These were allocated into 38 strata. Three large administrative regions (Moscow city, Moscow Region and St Petersburg city) constituted self-representing (SR) strata each. The remaining non-SR regions were allocated to 35 equal-sized strata. One region was then selected from each non-SR stratum using the probability proportional to size method.

The selection of second-stage units (SSUs) differed depending on whether the population of the specific PSU was urban, rural or mixed (cities or towns, urban settlements and villages). In urban PSU, SSUs were defined by the boundaries of census enumeration districts, voting districts or residential postal zones. In rural PSU, villages served as the SSUs. Within each mixed PSU, the population was stratified into urban and rural substrata, and the target sample size was allocated proportionately to the two substrata. In rural substrata, villages served as the SSUs; in urban substrata, SSUs were enumeration districts or voting districts.

Second-stage units (census enumeration districts, voting districts and villages) were selected from applicable lists systematically if SSUs were roughly equal in population size and with using probability proportional to size if they were unequal. The number of SSUs meets the requirement—10 selected dwellings in one SSU.

Dwellings in villages were selected systematically from existent lists of dwelling units. In urban survey districts (census enumeration and voting postal districts), the first step involved the enumeration of dwelling units by visual inspection; the second step involved systematic selection from organized dwelling units list.

The target RLMS sample size was set at 4000 dwelling units. To allow for the non-response, the sample actually amounted to 4718 dwelling units in 158 cities and towns, urban and rural settlements from 38 regions of the Russian Federation.

### Item Generation

The item generation from the ICF was carried out by an expert panel of the Institute of Nutrition of the Russian Academy of Medical Sciences and Vision International People Group, within preparation of the Russian National Population Quality of Life, Health and Nutrition Survey.

One of the modules included in the surveys was the nutritional status assessment of the respondents; therefore, items were selected from the ICF chapters that were likely to be sensitive to nutritional status and longitudinal change. In general, several items were chosen to represent each of the ICF major components: the Body Functions, the Activities and Participation, and the Environmental Factors. Chapters that cover body *structures* in the ICF were not included as they are less likely to be sensitive to change than body *functions*. The representative items for Body Functions were selected as sleep problems, emotional problems, eye problems, ear problems, pain, change in blood pressure, allergic reactions, heart problems, digestive problems, weight change, urinary problems, genito-sexual problems, musculoskeletal problems and skin problems to give a total of 18 items to represent Body Functions. Twenty-one items were selected to cover Activities and Participation and 21 items to cover Environmental Factors (seven items—Individual Environmental Factors; 14 items—Societal Environmental Factors). The overall HF-ICF-60 questionnaire consists of 60 items. The final HF-ICF-60 covers almost all chapters of the ICF components with the exception being the Voice and Speech Functions chapter of the Body Functions, and the Attitudes chapter of the Environmental Factors, because the items of these chapters were not the focus of the current study.

Both Body Functions and Activities and Participation were, following ICF guidelines, rated on five-point response scales that ranged from 1 = no problem to 5 = extreme problem. However, and again following ICF guidelines, the Environmental Factors were rated on nine-point scales from −4 to +4, the negative response points reflecting the fact that the item is a barrier (from ‘hinders mildly’ to ‘hinders completely’), with the positive responses indicating that the item is a facilitator (from ‘facilitates mildly’ to ‘facilitates completely’). For example, transport services could be rated as a facilitator for someone who is mobile and in good health, whereas the same services could be rated as a barrier for someone who is wheelchair-bound.

The list of items was generated in English initially before being translated into Russian so that the measure is now available in both languages. Both the items and the proposed response scales were discussed with staff of WHO, who are responsible for the development and maintenance of the ICF.

## STATISTICAL ANALYSIS

The approach for the statistical analyses was a combination of classical and modern psychometric approaches to questionnaire development. Following the earlier WHOQOL analytic guidelines (WHOQOL Group, 1998a, 1998b), descriptive statistics analysis examined item response frequency distributions, missing values analysis, internal and test–retest reliability, discriminant and construct validity, and sensitivity to change analyses, and exploratory factor analyses (EFA) and confirmatory factor analyses (CFA). In addition, an Item Response Theory (IRT) approach that used the Rasch model as implemented in the RUMM2030 program (Andrich, 2009) was also used. An iterative approach was taken in which the larger initial set of items was examined through a combination of classical and IRT approaches; thus, the earlier expert review from which the ICF items had been derived provided an initial possible structure for the items and which provided the starting point for the subsequent structural analyses of the HF-ICF-60 measure. Construct validity of the measure was assessed through correlation between the scales in the new measure and the WHOQOL-BREF domains, which participants also completed.

## RESULTS

The results from the HF-ICF-60 psychometric testing within the national population survey were available for two points in time, 1-year apart, for 2004 (Time 1) and for 2005 (Time 2). The decision was taken, therefore, to use the 2004 total dataset (*n* = 9807) for the descriptive statistics analyses, to use a random split-half of the 2004 data for running EFA of the measure and to use the second split-half of the 2004 data for running CFA but to carry out the IRT analyses on the 2005 total dataset (*n* = 9560). The existence of the repeated samples, 1-year apart, also allowed sensitivity to change comparisons, which are reported using Cohen’s *d* effect sizes.

### Descriptives

Table 2 shows the descriptive statistics for responses to each of the HF-ICF-60 items in the 2004 total dataset. Nearly all responses show <5.0% of missing values with the exception of item b4.3 (33.4%), a question about use of food supplements, which was not applicable for a third of the respondents (they did not take food supplements). The table shows minimum (=1) and maximum (=5) up to item b3.12 and maximum (=9) for the remaining items. For items up to b3.12, the mean values are low and close to the 1–2 range because of the fact that this is a primarily healthy nationally representative population completing a questionnaire designed to assess a degree of deviation in health status (the assessment ranges from absence of deviation [=1] to severe deviation [=5]). The table therefore shows some problems with skew and kurtosis for all of the items. If the descriptive statistics analyses are limited to those respondents who reported that they were ill, then the items show improved distributions and fewer problems with skew and kurtosis (Table 3).

**Table 2:** Descriptives for total sample, 2004 (*n* = 9807)

Item	*n*	% Missing	Min	Max	Mean	SD	Skewness	*p* (*z*-value)	Kurtosis	*p* (*z*-value)
b1.1	9796	0.1	1	5	2.22	1.095	0.428	<0.001	−0.916	<0.001
b1.2	9767	0.4	1	5	2.22	0.981	0.422	<0.001	−0.624	<0.001
b1.3	9740	0.7	1	5	1.53	0.878	1.651	<0.001	1.959	<0.001
b1.4	9766	0.4	1	5	1.63	0.947	1.432	<0.001	1.117	<0.001
b1.5	9748	0.6	1	5	1.90	0.984	0.883	<0.001	−0.068	ns
b1.6	9773	0.3	1	5	1.93	1.066	0.822	<0.001	−0.478	<0.001
b1.7	9747	0.6	1	5	1.31	0.719	2.521	<0.001	5.983	<0.001
b1.8	9781	0.3	1	5	1.73	0.981	1.207	<0.001	0.500	<0.001
b1.9	9760	0.5	1	5	1.34	0.733	2.346	<0.001	5.149	<0.001
b1.10	9609	2.0	1	5	1.50	0.815	1.649	<0.001	2.152	<0.001
b1.11	9775	0.3	1	5	1.68	0.889	1.243	<0.001	0.875	<0.001
b1.12	9797	0.1	1	5	1.36	0.783	2.339	<0.001	4.894	<0.001
b1.13	9775	0.3	1	5	1.28	0.673	2.654	<0.001	6.920	<0.001
b1.14	9464	3.5	1	5	1.41	0.800	2.139	<0.001	4.341	<0.001
b1.15	9773	0.3	1	5	1.70	0.996	1.270	<0.001	0.578	<0.001
b1.16	9735	0.7	1	5	1.59	0.949	1.524	<0.001	1.297	<0.001
b1.17	9773	0.3	1	5	1.59	0.922	1.518	<0.001	1.433	<0.001
b1.18	9780	0.3	1	5	1.40	0.762	2.046	<0.001	3.771	<0.001
b2.1	9789	0.2	1	5	1.80	0.895	0.889	<0.001	0.075	ns
b2.2	9771	0.4	1	5	1.87	0.938	0.850	<0.001	−0.054	ns
b2.3	9735	0.7	1	5	1.71	1.008	1.300	<0.001	0.813	<0.001
b2.4	9778	0.3	1	5	1.59	0.873	1.355	<0.001	1.070	<0.001
b2.5	9740	0.7	1	5	1.97	1.197	1.039	<0.001	−0.014	ns
b2.6	9649	1.6	1	5	2.26	1.431	0.742	<0.001	−0.874	<0.001
b2.7	9762	0.5	1	5	1.42	0.905	2.163	<0.001	3.740	<0.001
b2.8	9791	0.2	1	5	1.41	0.853	2.102	<0.001	3.585	<0.001
b2.9	9797	0.1	1	5	1.25	0.694	2.804	<0.001	7.167	<0.001
b3.1	9779	0.3	1	5	1.82	0.904	0.935	<0.001	0.312	<0.001
b3.2	9686	1.2	1	5	1.84	0.985	1.056	<0.001	0.478	<0.001
b3.3	9743	0.7	1	5	1.83	0.966	0.995	<0.001	0.274	<0.001
b3.4	9716	0.9	1	5	1.96	0.967	0.770	<0.001	−0.098	ns
b3.5	9695	1.1	1	5	2.05	1.009	0.674	<0.001	−0.380	<0.001
b3.6	9780	0.3	1	5	2.06	1.078	0.717	<0.001	−0.396	<0.001
b3.7	9790	0.2	1	5	1.81	0.907	0.824	<0.001	−0.206	<0.001
b3.8	9732	0.8	1	5	1.75	1.036	1.318	<0.001	1.006	<0.001
b3.9	9750	0.6	1	5	2.05	1.138	0.809	<0.001	−0.363	<0.001
b3.10	9680	1.3	1	5	2.06	1.305	0.972	<0.001	−0.280	<0.001
b3.11	9605	2.1	1	5	2.32	1.482	0.673	<0.001	−1.024	<0.001
b3.12	9525	2.9	1	5	2.51	1.529	0.471	<0.001	−1.271	<0.001
b4.1	9756	0.5	1	9	5.47	1.607	0.045	ns	0.410	<0.001
b4.2	9359	4.6	1	9	6.08	1.339	−0.414	<0.001	1.268	<0.001
b4.3	6529	33.4	1	9	5.36	1.159	0.150	<0.001	4.202	<0.001
b4.4	9745	0.6	1	9	4.69	1.702	0.229	<0.001	0.499	<0.001
b4.5	9761	0.5	1	9	4.70	1.380	0.210	<0.001	1.868	<0.001
b4.6	9711	1.0	1	9	6.12	1.753	−0.191	<0.001	−0.295	<0.001
b4.7	9717	0.9	1	9	5.95	1.404	0.219	<0.001	0.228	<0.001
b4.8	9712	1.0	1	9	4.27	1.612	−0.504	<0.001	0.519	<0.001
b4.9	9758	0.5	1	9	4.26	1.718	−0.124	<0.001	−0.055	ns
b4.10	9756	0.5	1	9	5.41	1.506	−0.033	ns	1.018	<0.001
b4.11	9742	0.7	1	9	5.07	1.469	−0.190	<0.001	1.519	<0.001
b4.12	9770	0.4	1	9	4.84	1.592	−0.381	<0.001	0.783	<0.001
b4.13	9696	1.1	1	9	4.82	1.237	−0.783	<0.001	3.451	<0.001
b4.14	9694	1.2	1	9	5.25	1.402	−0.217	<0.001	1.682	<0.001
b4.15	9750	0.6	1	9	4.70	1.710	−0.257	<0.001	0.204	<0.001
b4.16	9716	0.9	1	9	4.48	1.515	−0.625	<0.001	0.989	<0.001
b4.17	9759	0.5	1	9	4.50	1.584	−0.633	<0.001	0.806	<0.001
b4.18	9732	0.8	1	9	4.73	1.430	−0.743	<0.001	2.073	<0.001
b4.19	9786	0.2	1	9	4.88	1.617	−0.472	<0.001	0.694	<0.001
b4.20	9716	0.9	1	9	5.04	1.551	−0.295	<0.001	1.242	<0.001
b4.21	9738	0.7	1	9	4.39	1.555	−0.598	<0.001	0.552	<0.001

**Table 3:** Descriptives for ill sample, 2004 (*n* = 2167)

Item	*n*	% Missing	Min	Max	Mean	SD	Skewness	*p* (*z*-value)	Kurtosis	*p* (*z*-value)
b1.1	2166	0.0	1	5	2.84	1.121	−0.193	<0.001	−0.976	<0.001
b1.2	2159	0.4	1	5	2.68	1.024	−0.009	ns	−0.883	<0.001
b1.3	2151	0.7	1	5	1.98	1.099	0.798	<0.001	−0.536	<0.001
b1.4	2154	0.6	1	5	2.26	1.151	0.446	<0.001	−1.005	<0.001
b1.5	2146	1.0	1	5	2.65	1.085	0.022	ns	−0.948	<0.001
b1.6	2157	0.5	1	5	2.71	1.172	−0.115	ns	−1.177	<0.001
b1.7	2139	1.3	1	5	1.54	0.924	1.679	<0.001	1.820	<0.001
b1.8	2160	0.3	1	5	2.48	1.160	0.216	<0.001	−1.091	<0.001
b1.9	2142	1.2	1	5	1.66	0.988	1.352	<0.001	0.773	<0.001
b1.10	2092	3.5	1	5	1.72	0.941	1.098	<0.001	0.234	ns
b1.11	2161	0.3	1	5	2.09	1.057	0.629	<0.001	−0.636	<0.001
b1.12	2162	0.2	1	5	1.78	1.069	1.137	<0.001	0.119	ns
b1.13	2152	0.7	1	5	1.57	0.918	1.532	<0.001	1.393	<0.001
b1.14	2009	7.3	1	5	1.59	1.005	1.719	<0.001	2.062	<0.001
b1.15	2156	0.5	1	5	2.47	1.207	0.200	<0.001	−1.225	<0.001
b1.16	2141	1.2	1	5	2.29	1.218	0.380	<0.001	−1.187	<0.001
b1.17	2156	0.5	1	5	2.23	1.175	0.484	<0.001	−1.006	<0.001
b1.18	2155	0.6	1	5	1.78	1.020	1.113	<0.001	0.158	ns
b2.1	2161	0.3	1	5	2.18	0.995	0.460	<0.001	−0.564	<0.001
b2.2	2157	0.5	1	5	2.30	1.037	0.391	<0.001	−0.649	<0.001
b2.3	2148	0.9	1	5	2.29	1.191	0.527	<0.001	−0.736	<0.001
b2.4	2157	0.5	1	5	1.89	1.017	0.849	<0.001	−0.195	ns
b2.5	2154	0.6	1	5	2.88	1.390	0.067	ns	−1.289	<0.001
b2.6	2119	2.2	1	5	3.36	1.526	−0.400	<0.001	−1.341	<0.001
b2.7	2154	0.6	1	5	1.89	1.187	1.105	<0.001	0.074	ns
b2.8	2163	0.2	1	5	1.91	1.113	0.961	<0.001	−0.160	ns
b2.9	2162	0.2	1	5	1.42	0.819	1.862	<0.001	2.543	<0.001
b3.1	2157	0.5	1	5	2.38	1.034	0.387	<0.001	−0.456	<0.001
b3.2	2123	2.0	1	5	2.54	1.142	0.380	<0.001	−0.634	<0.001
b3.3	2144	1.1	1	5	2.42	1.098	0.396	<0.001	−0.575	<0.001
b3.4	2138	1.3	1	5	2.48	1.056	0.310	<0.001	−0.550	<0.001
b3.5	2131	1.7	1	5	2.60	1.085	0.141	ns	−0.832	<0.001
b3.6	2161	0.3	1	5	2.54	1.133	0.217	<0.001	−0.833	<0.001
b3.7	2162	0.2	1	5	2.01	0.970	0.502	<0.001	−0.772	<0.001
b3.8	2141	1.2	1	5	2.21	1.286	0.787	<0.001	−0.500	<0.001
b3.9	2148	0.9	1	5	2.37	1.280	0.466	<0.001	−0.975	<0.001
b3.10	2132	1.6	1	5	2.71	1.522	0.268	<0.001	−1.398	<0.001
b3.11	2116	2.4	1	5	3.10	1.613	−0.129	ns	−1.573	<0.001
b3.12	2101	3.0	1	5	3.12	1.613	−0.139	ns	−1.558	<0.001
b4.1	2149	0.8	1	9	5.05	1.602	−0.148	ns	0.535	<0.001
b4.2	2135	1.5	1	9	6.10	1.304	−0.763	<0.001	1.891	<0.001
b4.3	1304	39.8	1	9	5.27	1.164	−0.549	<0.001	4.526	<0.001
b4.4	2148	0.9	1	9	4.47	1.753	0.112	ns	0.071	ns
b4.5	2154	0.6	1	9	4.37	1.521	0.100	ns	0.776	<0.001
b4.6	2138	1.3	1	9	5.89	1.837	−0.222	<0.001	−0.268	ns
b4.7	2150	0.8	1	9	5.84	1.387	0.197	<0.001	0.404	<0.001
b4.8	2144	1.1	1	9	4.08	1.631	−0.577	<0.001	−0.134	ns
b4.9	2160	0.3	1	9	4.01	1.751	−0.123	ns	−0.513	<0.001
b4.10	2156	0.5	1	9	5.29	1.487	−0.147	ns	1.076	<0.001
b4.11	2144	1.1	1	9	4.88	1.406	−0.445	<0.001	1.726	<0.001
b4.12	2156	0.5	1	9	4.63	1.612	−0.407	<0.001	0.406	<0.001
b4.13	2127	1.8	1	9	4.65	1.249	−1.104	<0.001	2.556	<0.001
b4.14	2133	1.6	1	9	5.02	1.410	−0.363	<0.001	1.515	<0.001
b4.15	2153	0.6	1	9	4.29	1.706	−0.254	<0.001	−0.285	ns
b4.16	2135	1.5	1	9	4.26	1.529	−0.768	<0.001	0.328	ns
b4.17	2157	0.5	1	9	4.24	1.709	−0.493	<0.001	−0.152	ns
b4.18	2145	1.0	1	9	4.56	1.602	−0.647	<0.001	0.752	<0.001
b4.19	2161	0.3	1	9	4.60	1.799	−0.330	<0.001	−0.230	ns
b4.20	2125	1.9	1	9	4.80	1.419	−0.584	<0.001	1.831	<0.001
b4.21	2150	0.8	1	9	4.14	1.623	−0.590	<0.001	−0.232	ns

The responses of those who reported themselves to be ill also show <5.0% of missing values with the exception of item b1.14 (genito-sexual problems) and item b4.3 (use of food supplements).

### Exploratory Factor Analysis

The EFA was carried out on a random split-half of the 2004 sample, with later CFA being carried out on the other split-half of the 2004 sample. Both the EFA and the CFA have been run at the level of the four scales and will be reported as such. The EFA was run in SPSS Statistics 17.00 (SPSS Inc., 2008) using principal components analysis followed by varimax rotation. The EFA was run in order to provide some idea for defining the possible structure of the HF-ICF-60.

The EFA analyses for the Body Functions showed a three-factor solution for factors with Eigen values >1 that accounted for 55.4% of the variance, with Factor 1 (b1.15, b1.16, b1.17, b1.18) having highest loading item b1.16 (skeletal problems), Factor 2 (b1.1, b1.2, b1.3, b1.4, b1.5, b1.6, b1.8) with highest loading item b1.2 (emotional problems) and Factor 3 (b1.7, b1.9, b1.10, b1.11, b1.12, b1.13, b1.14) with highest loading item b1.13 (bladder problems).

The EFA for the Activities and Participation Scale gave a five-factor solution for Eigen values >1 that accounted for 70.5% of the variance, with Factor 1 (b2.5, b3.1, b3.2, b3.3, b3.4, b3.5) having highest loading item b3.2 (difficulty in doing the job), Factor 2 (b2.6, b3.10, b3.11, b3.12) with highest loading item b3.11 (difficulty with having leisure activities), Factor 3 (b2.7, b2.8, b2.9) with highest loading item b2.7 (difficulty in washing yourself), Factor 4 (b2.1, b2.2, b2.3, b2.4) with highest loading item b2.2 (difficulty in remembering or recollecting) and Factor 5 (b3.6, b3.7, b3.8, b3.9) with highest loading item b3.7 (difficulty in showing tolerance, respect and warmth in relationships).

The EFA for the Individual Environment Scale suggested a three-factor solution that accounted for 69.7% of the variance, but it should be noted that Factors 2 and 3 are not well formed in that both factors contain <3 items, the minimum number normally considered for a well-formed factor. The possibilities of 3-factor and 1-factor solutions were tested in the CFA and are described below. The EFA for the Social Environment Scale suggested a two-factor solution that accounted for 56.1% of the variance, with Factor 1 (b4.8, b4.15, b4.16, b4.17, b4.18, b4.19, b4.20, b4.21) having highest loading item b4.17 (social security services) and Factor 2 (b4.9, b4.10, b4.11, b4.12, b4.13, b4.14) having highest loading item b4.10 (communication services).

### Confirmatory Factor Analysis

The CFA was run on the second random split-half of the 2004 dataset using the EQS Version 6 program (Bentler & Wu, 2002), with the Robust Methods because of the non-normal distribution of data reported with the skew and kurtosis values in the Descriptives Section earlier.

For the 18-item Body Functions Scale, the CFA analyses show that the one-factor solution is a good starting point (CFI = 0.807, NFI = 0.802, Satorra–Bentler X^2^ = 4297.8, df = 135, *p* < 0.001, RMSEA = 0.079). An improved fit was obtained for a three-factor solution, found in the EFA, with a higher-order factor (CFI = 0.913, NFI = 0.908, Satorra–Bentler X^2^ = 2002.8, df = 132, *p* < 0.001, RMSEA = 0.054). However, as predicted under the ICF structure, the best fitting model is a five-factor solution with an overall higher-order factor (CFI = 0.931, NFI = 0.926, Satorra–Bentler X^2^ = 1605.5, df = 130, *p* < 0.001, RMSEA = 0.048).

For the 21-item Activities and Participation Scale, the one-factor solution is a poor fit (CFI = 0.675, NFI = 0.672, Satorra–Bentler X^2^ = 13500.9, df = 189, *p* < 0.001, RMSEA = 0.120). An improved fit was obtained for a five-factor solution, found in the EFA, with a higher-order factor (CFI = 0.867, NFI = 0.863, Satorra–Bentler X^2^ = 5646.0, df = 184, *p* < 0.001, RMSEA = 0.078).

The seven-item Individual Environment Scale gave a poor fit for a one-factor solution (CFI = 0.646, NFI = 0.644, Satorra–Bentler X^2^ = 1444.5, df = 14, *p* < 0.001, RMSEA = 0.144). A three-factor solution, as suggested by the EFA, showed an improvement in fit (CFI = 0.820, NFI = 0.817, Satorra–Bentler X^2^ = 742.2, df = 14, *p* < 0.001, RMSEA = 0.103), and this model was considerably improved with the addition of a higher-order factor (CFI = 0.920, NFI = 0.918, Satorra–Bentler X^2^ = 333.7, df = 11, *p* < 0.001, RMSEA = 0.077).

The 14-item Social Environment Scale showed that a one-factor solution provided a moderately good fit (CFI = 0.813, NFI = 0.809, Satorra–Bentler X^2^ = 3128.6, df = 77, *p* < 0.001, RMSEA = 0.090), but an improved fit was obtained for a two-factor solution, found in the EFA, with a higher-order factor (CFI = 0.875, NFI = 0.871, Satorra–Bentler X^2^ = 2112.0, df = 75, *p* < 0.001, RMSEA = 0.074).

Overall, the CFA supports the use of the four scales and also the use of total scores for each of these proposed scales, as suggested by a significantly improved fit for each scale when a higher-order factor is included in the structure. However, the analyses show that although fit improved through the inclusion of the higher-order factors that supported the use of a total scale score, nevertheless, the scales were not unidimensional in structure but were best described by models that contained two or more lower-order factors.

**Figure 1:**
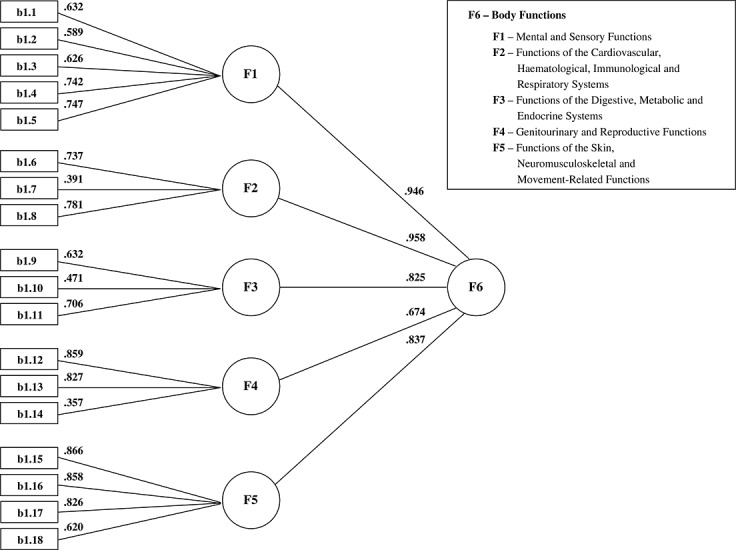
Best fitting Confirmatory Factor Analyses model for the Body Functions Scale

The above approach is used in the final structure of the Body Functions Scale, which comprises five subscales of three to five items each. This model includes an overall higher-order factor onto which all lower-order factors load (Figure 1). The reported model would suggest that the Body Functions Scale could be scored to give a total score in addition to a profile of scores across the five subscales.

For the rest of the HF-ICF-60 scales, i.e., the Activities and Participation, the Individual Environment and the Social Environment Scales, the total scores for each of the scales were used for the purpose of the current study. It should be noted also that all four scales of the HF-ICF-60 were positively correlated with each other for the full 2004 dataset, with Pearson r ranging from 0.148 (the Activities and Participation Scale with the Social Environment Scale) to 0.704 (the Body Functions Scale with the Activities and Participation Scale).

Because each of the HF-ICF-60 scales appeared to have more complex structures than a simple unidimensional model, the Rasch analyses are presented only in summary here. Detailed analyses of a short form of the HF-ICF-60 will be presented in more detail in a separate paper.

### IRT Analyses

The main purpose of the IRT analyses presented here was to check on item performance in terms of scale fit, plus Differential Item Functioning (DIF) in relation to men–women and healthy–ill respondent groups. The IRT approach used was that of the Rasch Unidimensional model approach, as instantiated in the RUMM2030 program (Andrich, 2009). Although the EFA and CFA had suggested that each of the four scales from the HF-ICF-60 might contain two or more subscales, the CFA results showed that nevertheless, each of the subscales contained one overall higher-order factor on which all of the items loaded significantly. Such complex factor structures mean that the unidimensional Rasch model analyses would be likely to show poor fitting items for each scale of the questionnaire because they are multidimensional and, indeed, the IRT analyses showed this to be the case. For the 2005 total sample, FitResid <3.0 was only obtained for 2/18 items of the Body Functions Scale, 5/21 items of the Activities and Participation Scale, 2/7 items of the Individual Environment Scale and for 3/14 items of the Social Environment Scale. A number of items with good fit properties increased for the ill sample, with FitResid <3.0 this time obtained for 11/18 items of the Body Functions Scale, 8/21 items of the Activities and Participation Scale, all seven items of the Individual Environment Scale and for 7/14 items of the Social Environment Scale.

Item reduction analyses were then carried out both on the basis of the Rasch properties of items and on the previous analyses, to develop a short form of the HF-ICF-60.

The main focus for the current analyses is on DIF of items, which indicates if an item performs differently between key groups (e.g., men versus women) even though individuals are at the same level of the underlying trait. Thus, DIF is useful for testing if an individual item is biassed to give higher or lower scores for those members of the target group, such as men versus women, even when those respondents have the same overall score for the total scale.

For the Body Functions Scale, the overall Person Separation Index (PSI) (the Rasch equivalent of the Cronbach alpha) is 0.827 for the total sample and 0.885 for those who reported themselves to be ill. Eleven of the items showed gender DIF and 16 showed health DIF for the total sample, but for the ill sample, only items b1.6 (change in blood pressure), b1.13 (bladder problems) and b1.14 (genito-sexual problems) showed gender DIF.

For the Activities and Participation Scale, PSI = 0.879 for the total sample and 0.923 for the ill sample. Ten items showed gender DIF, and 17 items showed health DIF for the total sample. For the ill sample, only item b2.6 (difficulty in running, jumping and swimming) showed gender DIF.

For the Individual Environment Scale, PSI = 0.753 for the total sample and 0.704 for the ill sample. Six items showed gender DIF, and four items showed health DIF for the total sample. For the ill sample, no items showed gender DIF.

For the Social Environment Scale, PSI = 0.909 for the total sample and 0.890 for the ill sample. Two items showed gender DIF, and seven items showed health DIF for the total sample. For the ill sample, no items showed gender DIF.

In summary, Rasch analyses indicated that all of the scales had one or more items that showed DIF for both gender and health for the total sample. However, item performance was substantially better for the ill sample with almost no items showing DIF.

### Internal and Test–Retest Reliability

The previous analyses have supported the use of the complete set of items for the HF-ICF-60, so in the remainder of the results, key characteristics of these now established scales will be presented. The Cronbach alpha values were computed for each of the scales of the HF-ICF-60. In the 2004 dataset, the Cronbach alpha values were 0.914 for the overall Body Functions Scale, 0.932 for the Activities and Participation Scale, 0.718 for the Individual Environment Scale and 0.912 for the Social Environment Scale. Similar alpha values were obtained for the 2005 dataset, and none of the corrected item-total correlations fell below 0.30 for either dataset.

The longitudinal design of the survey allows a test–retest correlational analysis of each item at the two time points. Of course, test–retest reliability for questionnaires such as the HF-ICF-60 is normally carried out over a 2- to 4-week period in order to avoid the impact of changes on the scores, so the retest period of approximately 12 months is longer than would normally be considered. Therefore, to minimize the impact of change, the test–retest correlations were assessed for the respondents who reported themselves to be healthy at both time points (*n* = 5100). The Body Functions (b1.1 − b1.18) item Pearson *r* values range from 0.260 for item b1.14 (genito-sexual problems) to 0.439 for item b1.6 (change in blood pressure), with the overall Body Functions Scale Pearson *r* = 0.546.

The item correlations for the Activities and Participation Scale (b2.1 to b2.9 − b3.1 to b3.12) range from 0.244 for item b3.9 (difficulty in solving problems connected with money) to 0.526 for item b2.6 (difficulty in running, jumping and swimming). The consistency for b2.6 may reflect consistent lifestyle choices that people make about their physical activity levels and which remain consistent over a 1-year period. The overall Activities and Participation Scale showed a 12-month correlation of 0.488.

The Individual Environment Scale (b4.1 − b4.7) shows Pearson *r* values that range from 0.280 for item b4.2 (use of drugs) to 0.421 for item b4.4 (outdoor air quality), with a scale total of 0.428. The variability for b4.2 may reflect the fact that this is a primarily healthy population with only occasional and short-term prescribed drug use. The Social Environment Scale (b4.8 − b4.21) shows Pearson *r* values that range from 0.209 for item b4.18 (social support services) to 0.334 for item b4.14 (media services) with the overall scale at 0.415. The items for this scale generally have low test–retest values, which may reflect the low and only occasional use of such services in a generally healthy population.

### Discriminant Validity and Sensitivity to Change

One of the discriminant group comparisons that can be made for the HF-ICF-60 items and scales is whether or not they distinguish between respondents who report themselves to be well versus respondents who report themselves to be ill. In fact, because of the longitudinal design, a group comparison can be made between those respondents who reported themselves to be well at both time points (*n* = 5100) versus those who reported themselves to be ill at both time points (*n* = 964). The item comparisons were nearly all significant (ts significant at *p* < 0.001 or better) apart from item b4.2 (use of drugs) from the Individual Environment Scale. Comparisons between the two groups were also significant for the four scales with the ill–ill group showing significantly lower scores than the well–well group (all ts at *p* < 0.001; Cohen’s *d* (using the formula where σ_pooled_ = √ [(n_1_ – 1)σ_1_^2^ + (n_2_ – 1)σ_2_^2^]/(n_1_ + n_2_ – 2)) ranged from 0.32, a small effect size, for the Individual Environment to 1.81, a large effect size, for Body Functions).

Additionally, a group comparison can be made between those respondents who reported themselves to be non-overweight with BMI <25 at both time points (*n* = 2762) versus those who reported themselves to be overweight with BMI ≥25 at both time points (*n* = 2619). Similarly to comparisons between well versus ill, the item comparisons were all significant (most ts significant at *p* < 0.001) apart from item b4.2 (use of drugs) and item b4.6 (relationships with members of the family) from the Individual Environment Scale. Comparisons between the two groups were also significant for the four scales with the overweight group with BMI ≥25 at both time points showing significantly lower scores than the non-overweight group with BMI <25 at both time points (all ts at *p* < 0.001; Cohen’s *d* [using the formula where σ_pooled_ = √ (σ_1_^2^ + σ_2_^2^)/2] ranged from 0.14, a small effect size, for the Individual Environment to 0.53, a medium effect size, for Body Functions).

A second set of analyses permitted by the longitudinal design is whether or not individual items and scales are sensitive to a change in the health status of respondents, e.g., for those who report that they are healthy in 2004 but ill in 2005 (*n* = 742). The paired *t*-tests for the Body Functions Scale showed all but item b1.1 (sleep problems) showed significant change and the overall Body Functions Scale itself also showed a significantly lower value at Time 2 (*p* < 0.001; *d* = 0.32). For the Activities and Participation Scale, all but item b2.2 (difficulty in remembering or recollecting) and item b3.7 (difficulty in showing tolerance, respect and warmth in relationships) showed significant changes, as did the overall scale (*p* < 0.001; *d* = 0.27). For the items of the Individual and Social Environment Scales, as would be predicted, fewer items (7/21) showed significant change with a change in health status between Time 1 and Time 2, with no change for the overall Individual Environment scores but a small increase (*p* < 0.05; *d* = 0.09) for the Social Environment.

Another set of analyses is whether or not the HF-ICF-60 individual items and scales are sensitive to a change in the nutritional status of respondents, e.g., for those who report that they are non-overweight (BMI <25) in 2004 but overweight (BMI ≥25) in 2005 (*n* = 410). The paired *t*-tests for the Body Functions Scale showed that six of 18 items showed significant change, e.g., digestive problems, urinary problems and weight change proper, as did the overall scale (*p* < 0.05; *d* = 0.10). For the Activities and Participation Scale, no items or the overall scale showed significant change. For the items of the Individual and Social Environment Scales, six of 21 items were sensitive to change with a change in nutritional status between Time 1 and Time 2, with no change for the overall Individual Environment scores but a small increase (*p* < 0.05; *d* = 0.10) for the Social Environment.

The reported psychometric properties were carefully tested to further develop a short form of the HF-ICF-60.

### Construct Validity

The administration of the WHOQOL-BREF (WHOQOL Group, 1998b) in the Russian National Population Quality of Life, Health and Nutrition Survey, as part of the RLMS, allowed a further validity test of the HF-ICF-60 scales through their correlation with domains from the WHOQOL-BREF. For example, for the 2004 dataset, the Pearson r correlation between the Body Functions Scale and the WHOQOL-BREF Physical Domain was 0.717, between the Activities and Participation Scale and the WHOQOL-BREF Psychological Domain was 0.585 and the Social Relations Domain 0.394, between the Individual Environment Scale and the WHOQOL-BREF Environment Domain was 0.390, and between the Social Environment Scale and the WHOQOL-BREF Environment Domain was 0.365 (all *p* < 0.001). Similar values were obtained for the 2005 dataset. The HF-ICF-60 shows good construct validity therefore when compared with a well-established measure such as the WHOQOL-BREF.

### Normative Values

The administration of the HF-ICF-60 and the WHOQOL-BREF in the Russian National Population Quality of Life, Health and Nutrition Survey carried out on a nationally representative sample from the RLMS has allowed the normative values for the Russian population to be obtained. Tables 4 and 5 show the population norms for the HF-ICF-60; Table 6 shows the normative values for the WHOQOL-BREF, obtained for the Russian population in 2005 (*n* = 9560). Higher health and functioning parameters were found in men and younger age groups, and lower health and functioning parameters were found in women and older age groups. The quality of life parameters demonstrate consistent results.

**Table 4:** Normative values for the Health and Functioning ICF-60 for the Russian population, 2005 (*n* = 9560)

			Body functions	Activities and participation	Individual environment	Social environment
Male	14–19	Mean	92.31	85.97	20.73	7.28
		SD	11.60	13.85	26.99	25.77
	20–29	Mean	92.78	86.17	15.43	0.93
		SD	9.72	13.67	24.50	23.59
	30–39	Mean	90.92	85.12	14.58	−3.27
		SD	10.47	13.61	23.29	25.30
	40–49	Mean	86.76	80.61	12.71	−5.70
		SD	13.38	15.24	25.16	28.03
	50–59	Mean	83.25	76.25	9.50	−10.38
		SD	14.04	16.30	22.67	27.74
	60–69	Mean	76.99	69.05	11.67	−9.07
		SD	16.30	19.36	24.67	29.46
	70+	Mean	72.39	60.47	15.48	−6.24
		SD	17.23	20.30	22.22	24.47
Female	14–19	Mean	90.64	86.46	18.37	8.04
		SD	10.45	12.69	25.33	21.81
	20–29	Mean	90.18	85.80	14.75	1.54
		SD	10.02	11.94	23.41	22.95
	30–39	Mean	86.85	82.82	13.28	−3.24
		SD	12.17	14.06	25.39	25.94
	40–49	Mean	82.18	78.51	9.67	−7.63
		SD	13.98	15.18	24.71	27.79
	50–59	Mean	78.41	73.93	9.72	−8.94
		SD	14.86	15.19	23.84	26.49
	60–69	Mean	72.58	66.46	11.50	−8.44
		SD	15.49	17.04	24.30	25.87
	70+	Mean	65.10	53.43	11.98	−2.84
		SD	17.32	19.32	21.05	21.19

**Table 5:** Normative values for the Health and Functioning ICF-60 (Body Functions Scale) for the Russian population, 2005 (*n* = 9560)

			Mental and sensory functions	Functions of the cardiovascular, haematological, immunological and respiratory systems	Functions of the digestive, metabolic and endocrine systems	Genitourinary and reproductive functions	Functions of the skin, neuromusculoskeletal and movement-related functions
Male	14–19	Mean	87.61	92.24	93.65	96.82	93.85
		SD	13.99	15.04	12.61	11.99	13.66
	20–29	Mean	87.47	93.40	93.31	97.17	95.17
		SD	13.13	12.20	11.55	10.40	11.68
	30–39	Mean	85.03	91.44	91.82	95.94	93.31
		SD	13.96	13.67	13.02	11.06	13.20
	40–49	Mean	80.48	86.80	89.72	93.52	87.45
		SD	16.95	16.33	14.26	13.84	17.90
	50–59	Mean	76.73	82.66	88.83	90.14	82.38
		SD	17.12	17.79	14.81	16.63	19.42
	60–69	Mean	70.68	76.36	84.98	82.49	75.16
		SD	20.19	17.96	17.09	20.54	23.14
	70+	Mean	68.12	73.66	80.87	76.63	67.16
		SD	17.88	19.36	20.61	24.93	24.34
Female	14–19	Mean	84.59	92.10	91.39	93.34	94.56
		SD	14.04	12.92	12.91	13.74	11.47
	20–29	Mean	84.01	91.24	89.60	93.16	95.29
		SD	13.82	13.05	14.12	11.73	10.57
	30–39	Mean	80.43	86.24	87.31	91.51	91.49
		SD	15.09	15.96	15.53	14.20	15.41
	40–49	Mean	74.83	81.03	84.83	89.39	84.82
		SD	17.46	17.60	16.88	15.38	19.01
	50–59	Mean	71.68	76.05	82.97	87.93	77.76
		SD	17.81	18.83	17.87	17.26	20.98
	60–69	Mean	65.15	68.62	80.63	84.34	70.48
		SD	18.47	19.01	19.12	19.37	22.12
	70+	Mean	56.93	63.65	73.96	79.53	58.94
		SD	19.43	19.16	21.77	22.20	24.88

**Table 6:** Normative values for the World Health Organization WHOQOL-BREF quality of life assessment for the Russian population, 2005 (*n* = 9560)

			Physical health and well-being	Psychological health and well-being	Social relations	Environmental factors	The total quality of life score
Male	14–19	Mean	81.26	71.60	71.76	62.37	71.75
		SD	14.00	13.64	17.80	14.81	11.82
	20–29	Mean	80.09	70.55	75.79	59.07	71.37
		SD	14.53	13.74	16.57	15.02	12.09
	30–39	Mean	75.49	66.12	72.07	56.70	67.58
		SD	15.55	13.78	18.28	14.52	12.65
	40–49	Mean	69.45	60.94	67.04	54.29	62.90
		SD	17.29	14.37	18.99	15.20	13.73
	50–59	Mean	64.30	57.90	63.13	53.10	59.60
		SD	18.80	14.69	19.54	15.50	14.44
	60–69	Mean	57.05	54.20	58.87	50.76	55.20
		SD	20.48	16.20	19.12	15.31	14.88
	70+	Mean	49.26	51.21	55.94	51.59	51.95
		SD	20.93	17.19	19.26	14.89	14.11
Female	14–19	Mean	78.39	69.90	71.21	60.46	70.03
		SD	14.59	14.32	19.52	15.27	12.58
	20–29	Mean	76.46	66.89	69.95	54.81	67.02
		SD	14.67	13.73	20.45	14.64	12.46
	30–39	Mean	71.29	61.89	65.95	52.08	62.79
		SD	15.81	14.48	19.99	14.57	13.05
	40–49	Mean	66.17	57.96	63.07	50.67	59.46
		SD	16.55	14.46	19.56	14.65	12.96
	50–59	Mean	60.48	54.68	59.70	49.25	56.04
		SD	17.49	14.34	18.34	14.40	12.82
	60–69	Mean	53.01	51.52	57.86	49.59	52.96
		SD	18.35	15.16	18.65	14.13	13.06
	70+	Mean	38.80	43.47	54.90	47.56	46.12
		SD	19.12	16.43	20.00	14.98	13.70

## DISCUSSION

The HF-ICF-60 measure reported in this paper was drawn from the ICF (WHO, 2001). The ICF was used in order to generate a measure that covered most of the key aspects of the ICF model that included Body Functions, Activities and Participation, and the Environmental Factors. The decision was taken therefore to focus on body functions rather than body structures because these are more likely to be sensitive to change in health and nutritional status.

The HF-ICF-60 measure consists of 60 items including 18 items that cover Body Functions, 21 items that cover Activities and Participation and 21 items that cover Environmental Factors (seven items cover Individual Environmental Factors and 14 items cover Societal Environmental Factors), which were selected from the ICF by an expert panel and corroborated by WHO. These items were considered to be most likely to be sensitive to change within the Russian National Population Quality of Life, Health and Nutrition Survey and included as part of the annual RLMS.

A range of psychometric tests shows that the separate scales and the overall questionnaire have good psychometric properties. Although most items had skew and kurtosis problems for the primarily healthy total sample, as expected, item distributions were much improved for the sub-sample of respondents who reported themselves to be ill.

A number of multivariate analyses were carried out that included EFA, CFA and IRT analysis using a Rasch model. The combination of EFA and CFA showed that although each of the four scales of the HF-ICF-60 may have an overall higher-order factor, they also have two or more lower-order factors as well. Such complex factor structures mean that the unidimensional Rasch model analyses would be likely to show poor fitting items for each scale of the questionnaire because they are multidimensional and, indeed, the IRT analyses showed this to be the case.

All multivariate analyses carried out showed good support for the proposed structure of the HF-ICF-60 questionnaire on the basis of the ICF model. Both EFA and CFA provided good support for the four-scale structure of the HF-ICF-60, which covers the ICF key components (WHO, 2001). Furthermore, the internal reliability Cronbach alpha values for the HF-ICF-60 scales were all high, and all of the corrected item-total correlations were above 0.30. Test–retest reliability is normally tested over a 2- to 4-week period, so the retest period of approximately 12 months is considerably longer than would normally be considered. Nevertheless, even at 12 months, there were satisfactory test–retest correlations for all the scales, because this is a primarily healthy sample, which remained healthy at both time points.

A number of further checks on the validity and sensitivity to change of the HF-ICF-60 measure were also carried out which included discriminant group (the respondents who reported themselves healthy versus those who reported themselves ill, and respondents who reported themselves non-overweight versus those who reported themselves overweight) comparisons; sensitivity to change from Time 1 to Time 2 for respondents who reported themselves to be well in Time 1 but became ill by Time 2, and for respondents who reported themselves to be non-overweight in Time 1 but became overweight by Time 2, and correlations with a well-known published measure, the WHOQOL-BREF (WHOQOL Group, 1998b), which assesses subjective quality of life. The tests demonstrated good discriminant and construct validity as well as sensitivity to change for the HF-ICF-60.

The normative values for the HF-ICF-60 obtained for the Russian population on a nationally representative sample from the RLMS have shown that higher health and functioning parameters were found in men and younger age groups, and lower health and functioning parameters were found in women and older age groups. Consistent results were obtained on the same sample for the normative quality of life values for the WHOQOL-BREF. Further analyses will be carried out with these data that will be reported in future papers.

In conclusion, the HF-ICF-60 questionnaire is a new measure derived directly from the ICF model of functioning and disability. The key components of the ICF model have formed the four main scales of the HF-ICF-60: namely, Body Functions, Activities and Participation, Individual Environment and Social Environment. The administration of the new measure in the two waves of the nationally representative RLMS has shown that the measure has good reliability and validity and that it is sensitive to change in the health and nutritional status of respondents over time. The high quality of the RLMS sample and good psychometric properties of the HF-ICF-60 allow the data to be used as the normative values for the Russian population. The administration of the WHOQOL-BREF in the nationally representative RLMS has also allowed the normative quality of life values for the Russian population to be obtained. The objective assessment of health and functioning of the HF-ICF-60 could be mapped onto the subjective evaluation of quality of life of the WHOQOL-BREF to increase the potential usefulness of the surveys in relation to non-communicable diseases. Therefore, the HF-ICF-60, a measure of health and functioning, can be recommended for use together with the WHOQOL-BREF, a quality of life measure, for national and global monitoring and surveillance of implementation of measures to reduce risk factors of non-communicable diseases and to promote healthy lifestyles and healthy diets.
